# Light‐Induced Disruption of 1D Wire‐Like Arrays of Monoatomic Ag(I) Ions: Single‐Crystal Reaction with Crystal Softening

**DOI:** 10.1002/anie.202419875

**Published:** 2025-05-02

**Authors:** Changan Li, Akalanka B. Ekanayake, Qianli R. Chu, Dale C. Swenson, Alexei V. Tivanski, Leonard R. MacGillivray

**Affiliations:** ^1^ Department of Chemistry University of Iowa Iowa City Iowa 52242 USA; ^2^ Département de Chimie Université de Sherbrooke Quebec J1K 2R1 Canada; ^3^ Department of Chemical Engineering Columbia University in the City of New York New York New York 10027 USA; ^4^ Department of Chemistry University of North Dakota Grand Forks North Dakota 58202 USA

**Keywords:** [2 + 2] photodimerization, Atomic force microscopy, Crystal engineering, Materials science, Metal‐metal interactions, Self‐assembly

## Abstract

The exploitation of noncovalent bonding in the solid state is attractive to generate one‐dimensional (1D) wire‐like assemblies of metals and uncover dynamic and physical properties of such intriguing structures. Herein, we describe a metal‐organic crystal based on Ag(I) ions that assemble to be organized into 1D wire‐like assemblies maintained by argentophilic interactions. UV‐light irradiation of the crystal composed of the 1D structures results in a single‐crystal‐to‐single‐crystal (SCSC) photodimerization that transforms the 1D periodic metal arrays to isolated metal dimers. The structural reconfiguration creates small voids in the crystal and the resulting solids exhibit a substantial increase in softness up to 60%.

## Graphical abstract

An Ag(I)‐based metal‐organic crystal featuring 1D wire‐like assemblies undergoes UV‐induced single‐crystal‐to‐single‐crystal photodimerization, transforming periodic metal arrays into dimers, creating voids, and decreasing crystal stiffness.

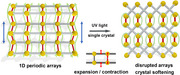

Ultra‐thin metal wire‐like assemblies, particularly structures that are linear and monoatomic, represent exemplary 1D systems with capacities to exhibit intriguing electrical, mechanical, magnetic, and chemical properties owing, in part, to quantum confinement of electrons.^[^ ^1^, ^2^, ^3^, ^4^, ^5^, ^6^, ^7^, ^8^, ^9^ ^]^ Despite unique characteristics, approaches to fabricate the materials remain challenging as a result of the Peierls distortion effect that relates to 1D structures being inherently unstable and tend to transition into more stable 2D or 3D forms.^[^ ^10^, ^11^, ^12^ ^]^ Even more lacking are examples that impart dynamic influences on the 1D structure, which can be critical to control electrical current (e.g., logic gates). Current synthetic methods for nanowires often rely on macromolecular templates such as DNA,^[^ ^13^, ^14^ ^]^ amino acids,^[^ ^15^, ^16^ ^]^ carbon nanotubes,^[^ ^17^, ^18^, ^19^ ^]^ and block copolymers,^[^ ^20^, ^21^, ^22^ ^]^ which typically operate on the nanometer scale and cannot provide structural control with atomic precision. While other approaches employ rigid organic and inorganic templates, such as calix[4]hydroquinone nanotubes and zeolites,^[^ ^23^, ^24^, ^25^ ^]^ to assemble metal wires with atomic accuracy, the materials generally lack the flexibility for structural transformation. Identifying crystalline structures that allow for structural changes of wires while also retaining single crystallinity, therefore, represents an unmet challenge and a significant gap in the understanding and development of nanowires with both atomic precision and dynamic control over structure.

Herein, we report the metal‐organic solid [**Ag**(**bpe**)][**CF_3_SO_3_**] [**1**; where **bpe **= *trans*‐1,2‐bis(4‐pyridyl)ethylene)] wherein Ag(I) ions are organized into 1D wire‐like assemblies sustained by argentophilic forces. We demonstrate **1** upon application of UV‐light to undergo a SCSC [2 + 2] photodimerization wherein conversion of the alkene to a cyclobutane in [**Ag(tpcb)_1/2_**][**CF_3_SO_3_**] (**2**; where **tpcb** = *rctt‐*tetrakis(4‐pyridyl)cyclobutane) disrupts the structure of the argentophilic forces. Specifically, the light imparts a dynamic influence on the 1D assemblies causing the Ag(I) ions to reorganize into isolated Ag(I) dimers (Scheme 1). The overall structural effect is a disruption of the continuous 1D periodic array that we demonstrate is accompanied by a dramatic increase in crystal softness up to 60%.

**Scheme 1:**
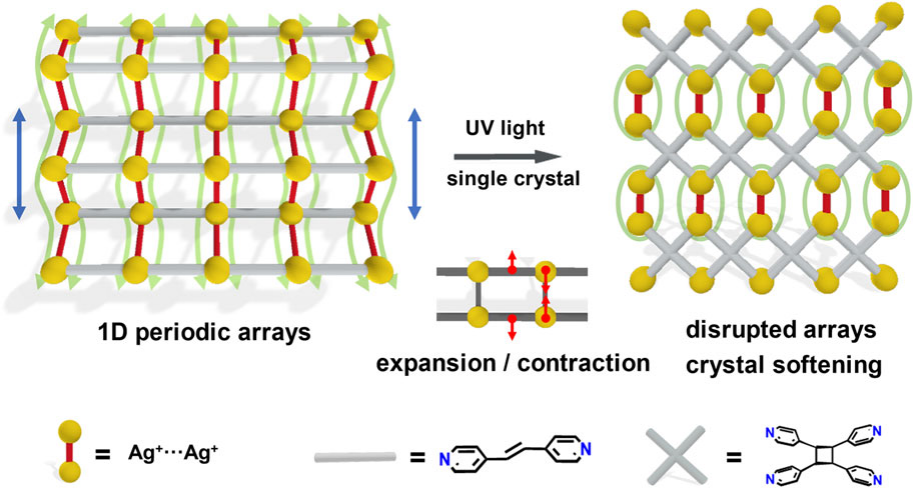
Light‐induced disruption of 1D arrays of argentophilic interactions.

Supramolecular chemistry and crystal engineering are emerging as new approaches for constructing well‐defined monoatomic nanowires.^[^ ^26^, ^27^, ^28^, ^29^, ^30^ ^]^ The incorporation of molecular components in a well‐defined environment allows for precise control through noncovalent bonding of the spatial arrangements of individual metals and related precursors (i.e., metal ions). Calix[4]hydroquinone has been used to assemble ultrathin silver nanowire arrays within channels sustained by van der Waals forces between the bowl‐shaped molecules.^[^ ^23^ ^]^ Tribenzocyclene derivatives were very recently reported to assemble monatomic nickel nanowires through hydrogen bonding.^[^ ^31^ ^]^ Despite advances in the use of noncovalent bonds to direct the assembly of metals into wire‐like structures in the crystalline state, there is a lack of reports on utilizing the approach to impart dynamic control over the 1D structures. We show here dynamic control of a 1D wire‐like structure that is achieved in a SCSC reaction, which provides an exceedingly unique perspective into the making and breaking of noncovalent forces in the material.

Metal‐organic crystal **1** was synthesized wherein Ag(I) precursors are organized into 1D wire‐like arrays by **bpe** ligand. When methanolic (2 mL) and acetonitrile (3 mL) solutions of **AgCF_3_SO_3_** (28.3 mg, 0.11 mmol) and **bpe** (18.2 mg, 0.1 mmol) were mixed, a white precipitate immediately formed. Subsequent heating to boil followed by sonication and filtration (0.22 µm PTFE membrane) with a syringe generated a solution from which micrometer‐sized crystals of **1** suitable for single crystal X‐ray diffraction (SCXRD) formed upon slow evaporation (3 d).

A SCXRD analysis revealed the components of **1** to crystallize in the triclinic space group *P*‐1 with **AgCF_3_SO_3_** and **bpe** in the asymmetric unit (Figure 1). The Ag(I) ions adopt a T‐shaped coordination geometry involving transoid Ag─N bonds (Ag─N1 2.141(4) Å, Ag─N2 2.143(4) Å; N1─Ag─N2 177.5(1)°) and a AgO bond (Ag···O2 2.765(4) Å) from **bpe** and the triflate ion, respectively. The components assemble to form a 1D coordination polymer along the crystallographic *bc*‐plane (Figure 1a). The polymers pack along the *a*‐axis to give a 2D layered structure sustained by face‐to‐face π···π stacking (centroid···centroid 3.754(2) Å) and argentophilic forces (Ag(I)···Ag(I) 3.784(3) and 3.838(2) Å). The argentophilic forces are manifested as 1D periodic arrays along the *a*‐axis (Figure 1b, green arrow). The formation of the 1D arrays of metal ions are in contrast to isolated Ag(I) dimers involving alkene‐based ligands.^[^ ^32^, ^33^, ^34^ ^]^ The strengths of the argentophilic forces alternate and are in the mid‐ to lower range.^[^ ^35^ ^]^ The 1D organization of the Ag(I) ions is akin to geometries of metal precursors in monoatomic metal wires (Figure 1c).^[^ ^36^ ^]^ The stacked C ═ C bonds are organized parallel and within 4.2 Å (3.824(6) and 3.967(7) Å), which conforms to the geometry criteria for a [2 + 2] photodimerization.^[^ ^37^ ^]^

**Figure 1:**
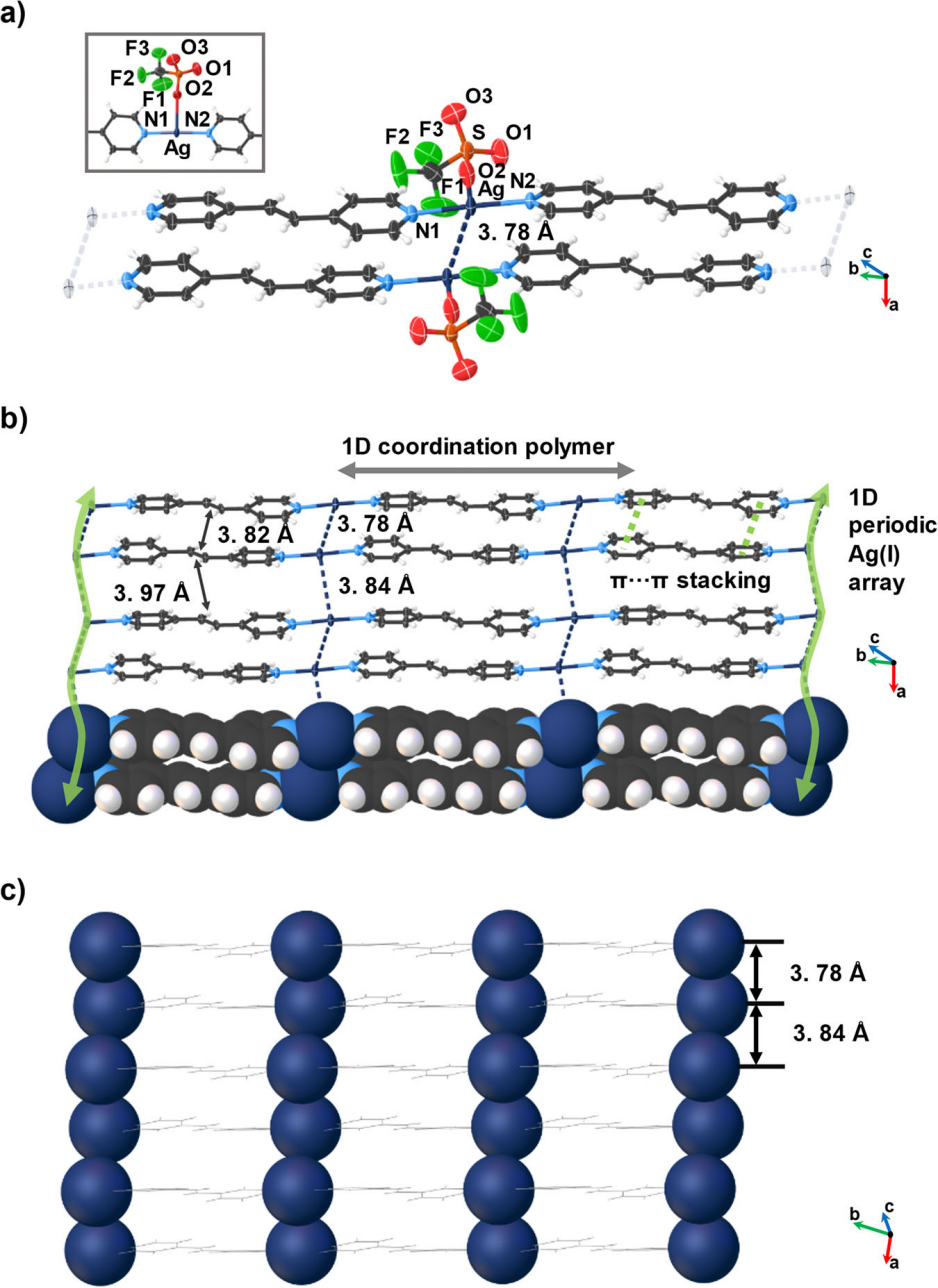
X‐ray structure **1**: a) coordination of Ag(I) ion, inset: coordination environment of the Ag(I) ion, b) extended stacking showing newly formed Ag(I)···Ag(I) interaction, and c) 1D periodic Ag(I)···Ag(I) arrays involving argentophilic forces. Counter ions omitted for clarity.

When a ground powder of **1** was irradiated with UV light (40 h, 450 W medium pressure Hg vapor lamp), **bpe** reacted to form **tpcb** in quantitative yield, as evidenced by the disappearance of the alkene peak (7.58 ppm) and appearance of a cyclobutane peak (4.66 ppm) in the ^1^H NMR spectrum (Figure S1). Optical microscopy showed that the single crystals remained intact during the photoreaction to generate **2**. A powder X‐ray diffractogram (PXRD) was consistent with the powder reacting to retain a crystal phase as **1** (Figures S2, S3).

A SCXRD analysis of **2** revealed **tpcb** to form quantitatively in a SCSC reaction (Figure 2). The Ag(I) ions retain the T‐shaped geometry with the Ag─N bonds (Ag─N1’ 2.131(6) Å, Ag─N2’ 2.123(6) Å; N1’─Ag─N2’ 169.9(2)°) and Ag─O bond (Ag─O1’ 2.680(6) Å) (Figure 2a, inset). The generation of **tpcb** was, moreover, accompanied by a significant disruption of the argentophilic forces. Specifically, the formation of the cyclobutane ring resulted in both breakage (Ag(I)···Ag(I) 4.41 Å) and reinforcement (Ag(I)···Ag(I) 3.15 Å) of the argentophilic forces. The result is the generation of Ag(I)···Ag(I) dimers that sit localized along the pathway of the original 1D arrays (Figure 2b). The breakage of the argentophilic forces within the wire‐like structures can be attributed to the pyridyl groups effectively “pushing” the Ag(I) ions away from each other to form the cyclobutane rings (Figure 2c).^[^ ^35^, ^38^, ^39^ ^]^ Two other structures involving Ag(I) ions and **tpcb** have been reported.^[^ ^40^, ^41^ ^]^ In both cases, the solids were, in contrast to **2**, obtained from solution with the Ag(I) ions being coordinated by four N‐atoms in a tetrahedral geometry. The lower coordination number of the metal atoms of **2** can, thus, be attributed to the inherently constrained nature of the SCSC reaction.^[^ ^42^ ^]^ Overall, the structural changes result in the volume of the unit cell increasing on the order of 2% (i.e., 740 Å^3^ to 754 Å^3^) (Tables S1, S2). Collectively, the application of the UV‐light resulted in a major redistribution of the Ag(I) ions within the solid.

**Figure 2:**
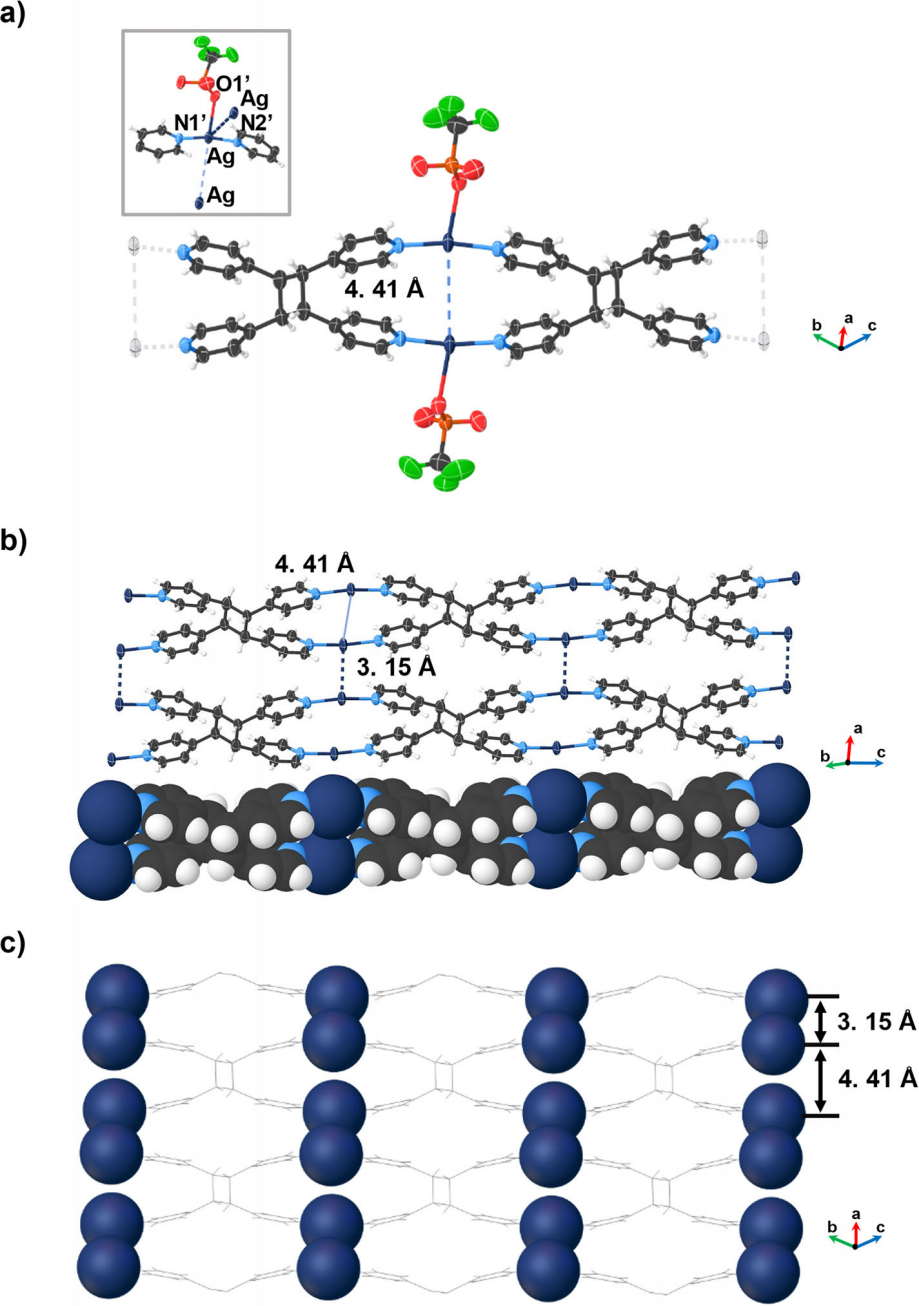
X‐ray structure of **2**: a) ORTEP view, inset: coordination environment of the Ag(I) ion, b) extended stacking showing the formation of new Ag(I)···Ag(I) interaction, and c) disrupted Ag···Ag arrays involving argentophilic forces. Counter ions omitted for clarity.

The photodimerization resulted in significant softening of the single crystals. Atomic force microscopy (AFM) nanoindentation revealed crystals of both micrometer and nanometer dimensions, which are of prism‐like morphologies (Figure 3a–d), to become softer (i.e., less stiff) following the photoreaction.^[^ ^43^ ^]^ The average Young's moduli decreased by ca. 60% (from 190 ± 30 MPa to 75 ± 30 MPa) and ca. 30% (from 180 ± 50 MPa to 130 ± 30 MPa) for the nano‐ and micro‐sized crystals, respectively (Figure 3e),^[^ ^44^, ^45^ ^]^ with differences between the moduli for both crystal sizes being confirmed using unpaired t‐tests (*P* < 0.05 for nano‐ and micro‐crystal). The decrease is comparable to the solid based on Ag(I) dimers (see also Table S3).^[^ ^34^ ^]^ Uncertainties in the reported average Young's moduli correspond to one standard deviation and may stem from different exposed crystallographic planes.^[^ ^46^, ^47^ ^]^

**Figure 3:**
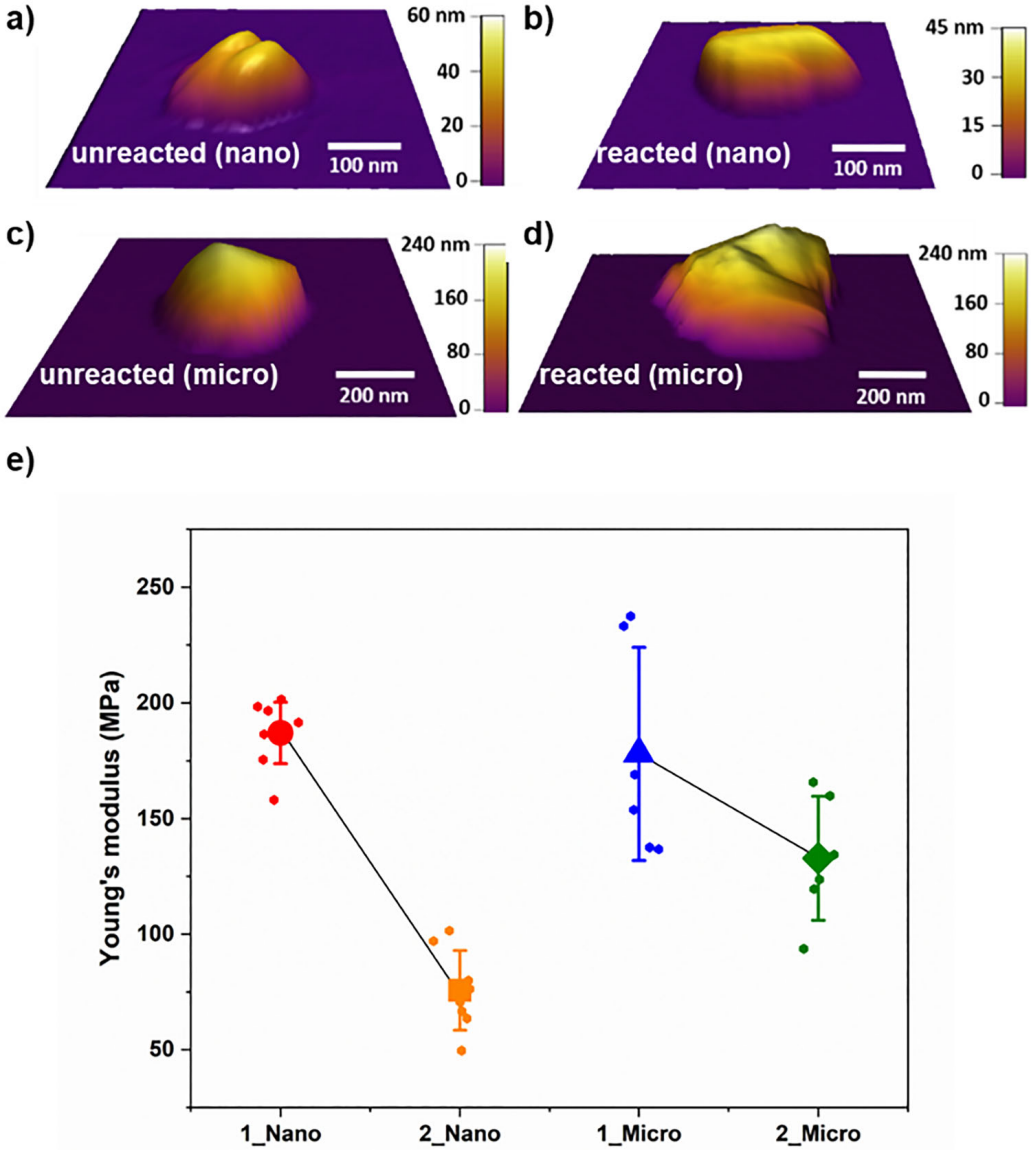
AFM 3D height images of individual crystals (before/after reaction): a) **1** nano (before), b) **2** nano (after), c) **1** micro (before), d) **2** micro (after), and e) Young's modulus values for individual crystals of **1** and **2** in nano‐ and micro‐dimensions. Error bars represent one standard deviation and no apparent changes were generally observed to surfaces of crystals as a result of the photoreaction.

The origin of the increase in softness of the single crystals upon photoreaction from **1** to **2** may be attributed to generation of void space resulting from the photoreaction in the solid. Void calculations show the 2% increase in unit cell volume to correspond to a 7% increase in accessible void space (i.e., 31.3% in **1** to 33.6% in **2** or decrease in density). The voids are located in areas between the reacting pyridyl groups of **2** associated with a cyclobutane ring (Figure 4a, red). The voids, thus, arise near the reacting C‐atoms that undergo conversion from sp^2^ to sp^3^ hybridization. Similar changes in Young's moduli have been reported when free space is modified for related porous metal‐organic frameworks (e.g., MOF‐74).^[^ ^48^, ^49^ ^]^ Half of the argentophilic forces are also lost upon reaction of **1** to **2**, albeit with the formation of the Ag(I) dimers and C─C single bonds of the cyclobutanes (Figure 4b).^[^ ^50^, ^51^ ^]^ Overall, the Ag(I) ions move nominally closer (i.e., contract 7.62 Å to 7.56 Å and 13.67 Å to 13.41 Å) while the cyclobutanes become appreciably more separated compared to the alkenes (i.e., expand 6.66 Å to 7.03 Å) within a layer (Figure 4b).^[^ ^52^, ^53^ ^]^ These movements may additionally give rise to generation of larger relative number of defects in the solid that may account for the increase in softness.^[^ ^54^, ^55^ ^]^ We are currently investigating those factors responsible for the change in mechanical properties of the material.

**Figure 4:**
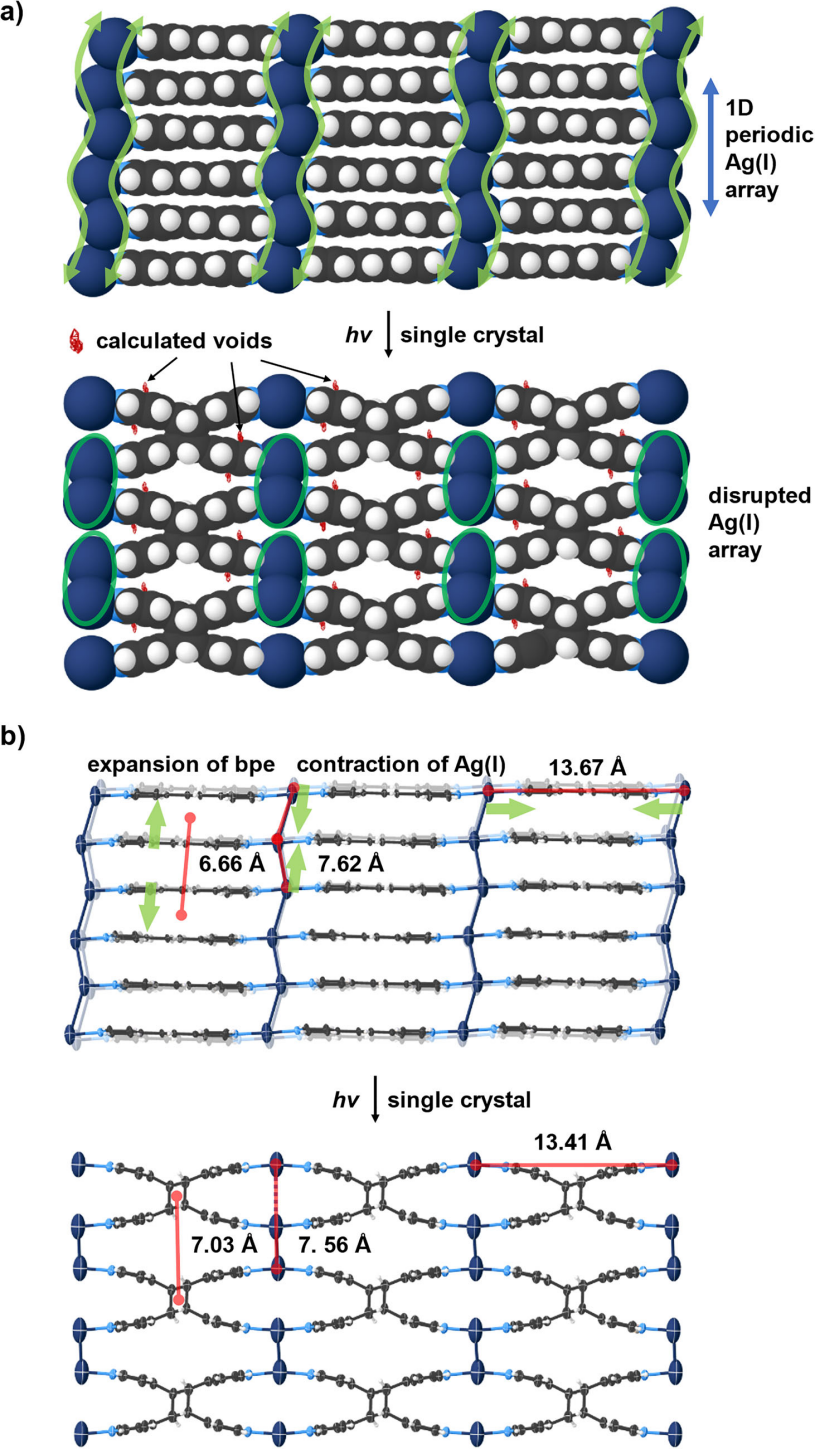
a) Structural transformation illustrating the redistribution of Ag(I) ions; free space generated by UV irradiation is highlighted as red wireframes, and b) light‐induced positional anisotropy of the Ag(I) and **bpe** ligand in the crystal lattice. Counter ions omitted for clarity.

In conclusion, we report a photoactive metal‐organic crystalline solid with Ag(I) ions assembled as 1D wire‐like monoatomic arrays. The UV irradiation transforms the wire‐like arrays into isolated Ag(I) dimers in a SCSC reaction that is accompanied by significant crystal softening. Such changes to mechanical properties in SCSC transformations can be crucial to better understand mechanisms that drive responses to light,^[^ ^56^, ^57^ ^]^ including surface morphology and the photosalient effects.^[^ ^58^, ^59^, ^60^, ^61^, ^62^ ^]^ We expect our findings to impact avenues to generate responsive crystalline materials composed of 1D atomic arrays as a means to implement dynamic control of solids. Our finding may also be foundational for future explorations wherein a transformation of 1D wires to dimers is exploited as a mechanism to further affect physical properties of the materials.

## Conflict of Interests

The authors declare no conflict of interest.

## Supporting information



Supporting Information

## Data Availability

The data that support the findings of this study are available in the supplementary material of this article.
