# Supplementary material for: Light‐Induced Disruption of 1D Wire‐Like Arrays of Monoatomic Ag(I) Ions: Single‐Crystal Reaction with Crystal Softening
Source: Angew Chem Int Ed Engl. 2025 May 2;64(26):e202419875. doi: 10.1002/anie.202419875 (PMC12184307; doi:10.1002/anie.202419875)
Supplement: Supplementary file 1 — Supporting Information [file ANIE-64-e202419875-s001.docx]

Supporting Information
©Wiley-VCH 2024
69451 Weinheim, Germany

Light-Induced Disruption of 1D Wire-Like Arrays of Monoatomic Ag(I) Ions: Single-Crystal Reaction with Crystal Softening

Changan Li, Akalanka B. Ekanayake, Qianli R. Chu, Dale C. Swenson, Alexei V. Tivanski* and Leonard R. MacGillivray *

**Abstract:** The exploitation of noncovalent bonding in the solid state is attractive to generate one-dimensional (1D) wire-like assemblies of metals and uncover dynamic and physical properties of such intriguing structures. Herein, we describe a metal-organic crystal based on Ag(I) ions that assemble to be organized into 1D wire-like assemblies maintained by argentophilic interactions. UV-light irradiation of the crystal composed of the 1D structures results in a single-crystal-to-single-crystal (SCSC) photodimerization that transforms the 1D periodic metal arrays to isolated metal dimers. The structural reconfiguration creates small voids in the crystal and the resulting solids exhibit a substantial increase in softness up to 60%.

DOI: 10.1002/anie.2021XXX

**Table of Contents**

**S1) Experimental Information**3

1. Materials3
2. Syntheses3
3. Solid-State Photoreaction3

(D) Atomic Force Microscopy (AFM) Measurements3

**S2) Single-Crystal X-Ray Diffraction (SCXRD) Data** 4

**S3) Nuclear Magnetic Resonance (NMR) Spectral Data** 8

**S4) Powder X-Ray Diffraction (PXRD) Data** 9

S1) Experimental Information

**A) Materials**:

All starting materials and solvents were used as received from commercial sources. *trans*-1,2-Bis(4-pyridyl)ethylene) (**4,4’-bpe**) and silver trifluoromethanesulfonate (**AgCF_3_SO_3_**) were obtained from Sigma-Aldrich. All solvents were purchased from Fischer Scientific.

**B) Syntheses**:

1) Synthesis of **[Ag(4,4’-bpe)][CF_3_SO_3_]** (**1**)

A methanol solution (2 mL) of **AgCF_3_SO_3_** (20 mg, 0.14 mmol) was added dropwise to an acetonitrile solution (3 mL) of **4,4'-bpe** (18.2 mg, 0.1 mmol) in a 1.1:1 molar ratio. A white precipitate formed immediately upon mixing. The mixture was then heated to boiling using a heat gun to ensure complete dissolution of the precipitate. The resulting solution was filtered through a 0.22 *μ*m PTFE syringe membrane. Micrometer-sized single crystals were obtained by slow evaporation over a period of approximately 3 days.

2) Synthesis of nanocrystals of **1**

The nano-cocrystals of **1** were prepared using a previously reported method involving precipitation combined with sonochemistry.^[1]^ **4,4’-bpe** and **AgCF_3_SO_3_** were separately dissolved in minimal ethanol and simultaneously injected into hexanes under ultrasonic irradiation for a period of 2 min. The resulting white powder was then washed with cold hexanes under vacuum.

**C) Solid-State Photoreaction**

Photodimerization reactions were carried out in an ACE Glass photochemistry cabinet fitted with a 450 W Hanovia medium-pressure mercury lamp, which emits across a broad wavelength range. Approximately 40-48% of the total energy lies in the UV region, 40-43% in the visible range, and the rest in the IR. To prepare the samples, single crystals of compound 1 were finely ground with a mortar and pestle, then spread between two Pyrex glass plates to ensure even exposure. The samples were irradiated in 8-hour intervals to allow for uniform light exposure, and the progress of the photodimerization to form *rctt*-tetrakis(4-pyridyl)cyclobutane (tpcb) was monitored by ^1^H NMR spectroscopy. For the single-crystal-to-single-crystal (SCSC) transformation, intact single crystals of compound 1 were irradiated in a UV light gel nail dryer (36 W) with a wavelength range of 380-480 nm, peaking at 365 nm, to provide controlled and consistent irradiation conditions.

D) Atomic Force Microscopy (AFM) Measurements

We investigated both micrometer- and nanometer-sized crystals of unreacted compound 1 and photoproduct 2. AFM imaging of unreacted and photoreacted solids 1 and 2 revealed the presence of individual crystals all exhibiting similar prism-like morphologies. For each sample, at least 6 individual crystals were characterized. To mitigate possible size effects, similar-sized crystals were studied for each sample type before and after photodimerization to ensure that any observed differences in the Young’s modulus can largely be attributed to the effects of photodimerization. The base size and height range were ca. 100-300 nm and 40-100 nm for nano-sized 1 and 2, and ca. 200-700 nm and 100-350 nm for micro-sized 1 and 2, respectively.

Crystals of **1** and **2** were suspended in methanol (1 mg in 10 mL), then drop-casted on a freshly cleaved atomically flat mica substrate (V-1 grade, SPI Supplies, Westchester, PA) and solvent was allowed to evaporate. All AFM studies were conducted using a Molecular Force Probe 3D AFM (Asylum Research, Santa Barbara, CA). AFM imaging and nanoindentation measurements were performed at room temperature and ambient pressure using Si_3_N_4_ probes (Mikromasch, San Jose, CA) with a nominal spring constant ranging from 2 N/m to 5.4 N/m, and a typical tip radius of curvature of 15 nm. Actual spring constants were determined using a built-in thermal noise method.^[2]^ Topographic AFM images (height and amplitude) were collected using an intermittent contact mode (AC mode) at a typical scan rate of 1 Hz.

AFM nanoindentation experiments were performed by recording force versus vertical piezo displacement (i.e., force curves) to determine Young’s modulus values of individual substrate-deposited micro- and nano-sized unreacted and photoreacted crystals of **1** and **2**. The Young’s modulus was determined by fitting the loading force versus indentation distance approach to the crystal surface data to the Johnson-Kendall-Roberts (JKR) contact model.^[3]^ The JKR model was selected due to close overlap between the approach and retract contact region data, confirming purely elastic nanoindentation, and the presence of adhesion force between the AFM tip and crystal surface. The acquisition of the force curves and corresponding data analysis was carried out as reported previously.^[4]^ For each individual crystal, typically 3-5 individual force curves were collected. The reported Young’s modulus for each sample type corresponds to the average and one standard deviation of values measured on multiple individual crystals.

S2) Single-Crystal X-Ray Diffraction (SCXRD) Data

Single-crystal X-ray diffraction (SCXRD) data of compound 1 were collected on a Bruker Nonius KappaCCD single-crystal X-ray diffractometer using MoKα radiation (λ = 0.71073 Å), graphite monochromator equipped with an Oxford Cryostream low temperature device. Data for compound 2 were collected on a Bruker Nonius APEX II Kappa single-crystal X-ray diffractometer using MoKα radiation (λ=0.71073 Å). Crystals were mounted in Paratone oil on a Mitegen magnetic mount. Lorentz and polarization corrections were applied and programs from the APEXII package were used for data reduction. Structure solution and refinement were performed using SHELXL ^[5]^ and SHELXT^[6]^, respectively within the Olex2^[7]^ graphical user interfaces. Crystallographic data are summarized in Tables S1 and S2.

**Table S1.** Crystallographic parameters for **[Ag(4,4’-bpe)][CF_3_SO_3_]** (**1**) .

| Compound name | **[Ag(4,4’-bpe)][CF_3_SO_3_]** |
| --- | --- |
| CCDC deposition number | 2390435 |
| Empirical formula | C_13_H_10_AgF_3_N_2_O_3_S |
| Formula weight | 439.16 |
| Temperature/K | 293(2) |
| Crystal system | triclinic |
| Space group | P-1 |
| a/Å | 6.662(5) |
| b/Å | 10.999(5) |
| c/Å | 11.039(5) |
| α/° | 95.516(5) |
| β/° | 103.220(5) |
| γ/° | 107.222(5) |
| Volume/Å^3^ | 740.4(7) |
| Z | 2 |
| ρ_calc_g/cm^3^ | 1.970 |
| μ/mm^‑1^ | 1.550 |
| F(000) | 432.0 |
| Crystal size/mm^3^ | 0.22 × 0.185 × 0.125 |
| Radiation | MoKα (λ = 0.71069) |
| 2Θ range for data collection/° | 5.974 to 49.99 |
| Index ranges | -7 ≤ h ≤ 7, -13 ≤ k ≤ 13, -13 ≤ l ≤ 13 |
| Reflections collected | 4267 |
| Independent reflections | 2502 [R_int_ = 0.0491, R_sigma_ = 0.0679] |
| Data/restraints/parameters | 2502/0/208 |
| Goodness-of-fit on F^2^ | 1.035 |
| Final R indexes [I>=2σ (I)] | R_1_ = 0.0451, wR_2_ = 0.1142 |
| Final R indexes [all data] | R_1_ = 0.0569, wR_2_ = 0.1222 |
| Largest diff. peak/hole / e Å^-3^ | 0.55/-0.81 |

**Table S2.** Crystallographic parameters for **[Ag(tpcb)_1/2_][CF_3_SO_3_]** (**2**) .

| Compound name | **[Ag(tpcb)_1/2_][CF_3_SO_3_]** |
| --- | --- |
| CCDC deposition number | 2390436 |
| Empirical formula | C_13_H_10_AgF_3_N_2_O_3_S |
| Formula weight | 439.17 |
| Temperature/K | 190.15 |
| Crystal system | triclinic |
| Space group | P-1 |
| a/Å | 7.033(5) |
| b/Å | 10.833(5) |
| c/Å | 10.875(5) |
| α/° | 76.293(5) |
| β/° | 73.572(5) |
| γ/° | 74.733(5) |
| Volume/Å^3^ | 754.8(7) |
| Z | 2 |
| ρ_calc_g/cm^3^ | 1.9322 |
| μ/mm^‑1^ | 1.521 |
| F(000) | 430.7 |
| Crystal size/mm^3^ | 0.04× 0.04× 0.24 |
| Radiation | Mo Kα (λ = 0.7107) |
| 2Θ range for data collection/° | 6.08 to 50 |
| Index ranges | -9 ≤ h ≤ 9, -14 ≤ k ≤ 14, -14 ≤ l ≤ 13 |
| Reflections collected | 6202 |
| Independent reflections | 2625 [R_int_ = 0.0716, R_sigma_ = 0.1196] |
| Data/restraints/parameters | 2625/0/208 |
| Goodness-of-fit on F^2^ | 1.045 |
| Final R indexes [I>=2σ (I)] | R_1_ = 0.0605, wR_2_ = 0.1386 |
| Final R indexes [all data] | R_1_ = 0.0974, wR_2_ = 0.1560 |
| Largest diff. peak/hole / e Å^-3^ | 1.94/-0.89 |

The total void space in the crystal structure was calculated in Olex2 with resolution defined as 0.2 Å.

Cell volume of 1: 740.378 Å^^3^

Radius [volume] of the largest spherical void is 1.00 Å [4.19 Å^^3^]

The void center(s) are at (fractional):

0.216 0.472 0.074

0.379 0.565 0.191

0.621 0.435 0.809

0.784 0.528 0.926

structure occupies: 508.45 Å^^3^ (68.67%)

Cell volume of 2: 754.765 Å^^3^

Radius [volume] of the largest spherical void is 1.20 Å [7.24 Å^^3^]

The void center(s) are at (fractional):

0.297 0.037 0.474

0.703 0.963 0.526

structure occupies (Å^^3^) 500.77 (66.35%)

S3) Nuclear Magnetic Resonance (NMR) Spectral Data


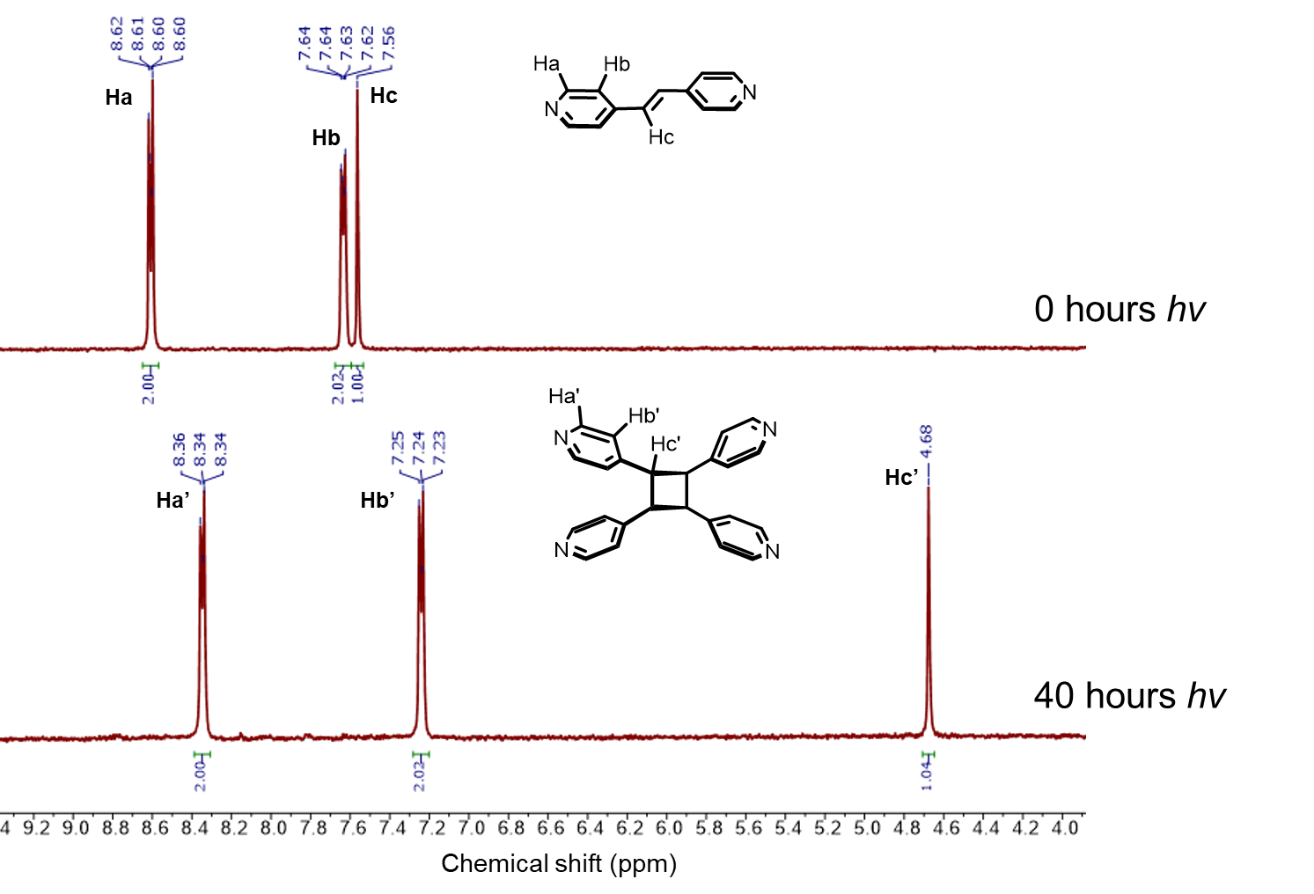
^1^H NMR spectra were collected at ambient temperature using Bruker AVANCE NEO 400 MHz NMR spectrometer. DMSO-*d*_6_ was used as NMR solvents. All NMR data were processed with MestreNova software suite.

Figure S1. ^1^H NMR spectra of 1 before (top) and after (bottom) UV irradiation for 40 hours (400 MHz, DMSO-*d*_6_).

S4) Powder X-Ray Diffraction (PXRD) Data


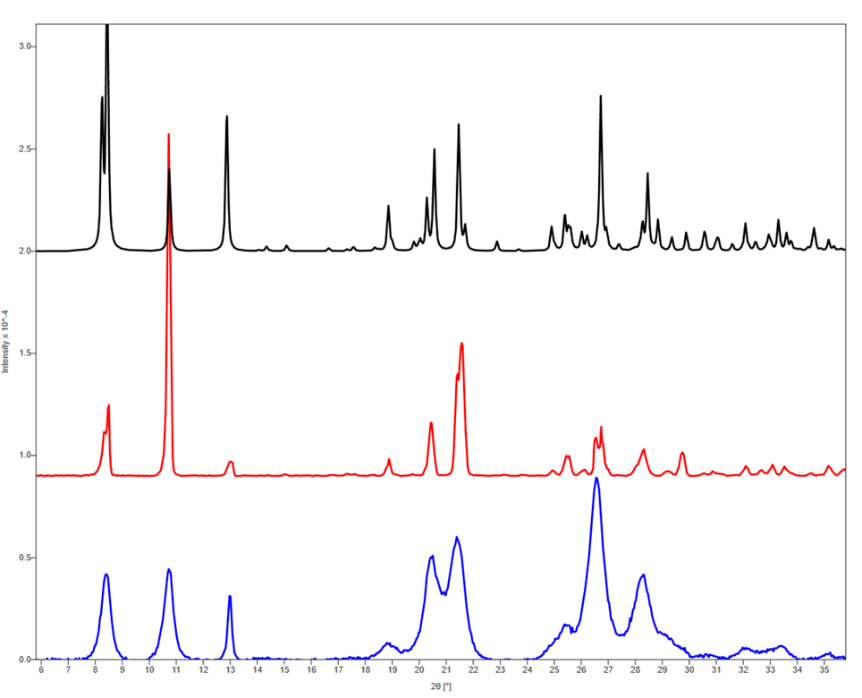
Powder X-ray diffraction data were collected at room temperature using a Bruker D8 Advance X-ray diffractometer with CuKα_1_ radiation (λ = 1.54056 Å) typically in the range of 5−36° two-theta (scan type: locked coupled; scan mode: continuous; step size: 0.02°). Samples were ground and mounted on glass slides. The equipment was operated at 40 kV and 30 mA.

**Figure S2**. Powder XRD patterns of **1**: simulated pattern from single crystal X-ray diffraction data (black), experimental pattern from powder sample of micro-sized crystals (red), and experimental pattern from powder sample of nano-sized crystals (blue).


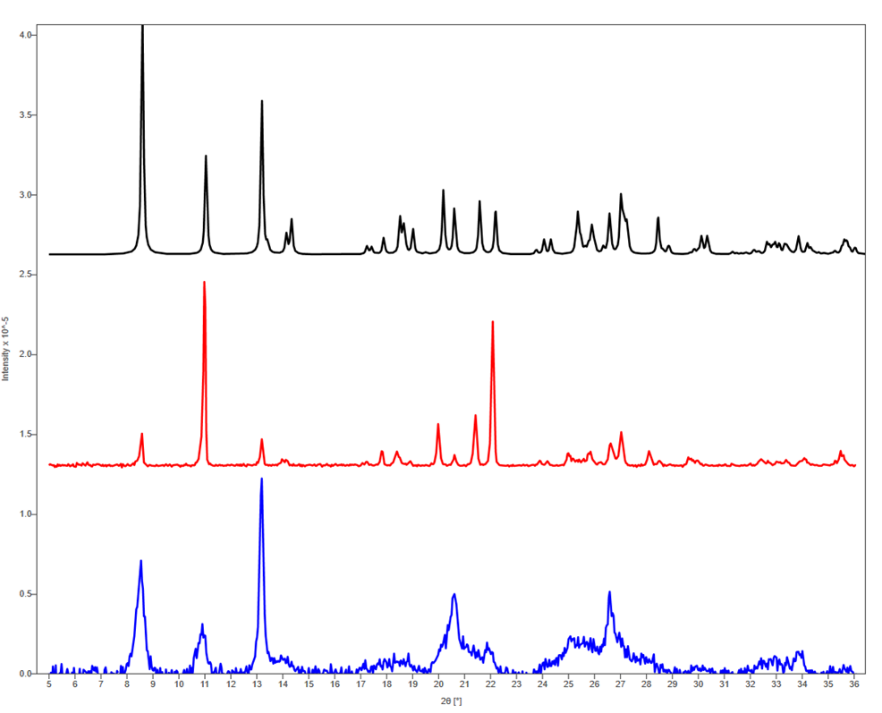
**Figure S3**. Powder XRD patterns of **2**: simulated pattern from single crystal X-ray diffraction data (black), experimental pattern from powder sample of micro-sized crystals (red), and experimental pattern from powder sample of nano-sized crystals (blue).

**Table S3.** Comparison of Young's modulus in crystals undergoing light-induced photodimerization.

| Literature reports | Crystal composition | YM before reaction | YM after reaction | Changes in YM |
| --- | --- | --- | --- | --- |
| This report | **[Ag(bpe)][CF_3_SO_3_]**  **bpe** = *trans*-1,2-bis(4-pyridyl)ethylene) | E_(micro)_ = 180 ± 45 MPa  E_(nano)_ = 190 ± 15 MPa | E_(micro)_ = 130 ± 30 MPa  E_(nano)_ = 75 ± 15 MPa | -25% micro  -60% nano |
| Hutchins *et al.*^[1]^ | **[Ag_2_(4-stilbz)_4_][CF_3_SO_3_]_2_**  **4-stilbz** = *trans*-4-styrylpyridine | E_(nano)_ = 505 ± 85 MPa | E_(nano)_ = 305 ± 50 MPa | -40% nano |

# References

[1] K. M. Hutchins, T. P. Rupasinghe, L. R. Ditzler, D. C. Swenson, J. R. Sander, J. Baltrusaitis, A. V. Tivanski, L. R. MacGillivray, *J. Am. Chem. Soc.* **2014**, *136*, 6778-6781.

[2] J. L. Hutter, J. Bechhoefer, *Rev. Sci. Instrum.* **1993**, *64*, 1868-1873.

[3] K. L. Johnson, K. Kendall, a. Roberts, *Proceedings of the royal society of London. A. mathematical and physical sciences* **1971**, *324*, 301-313.

[4] a) A. Tiba, J. Perman, L. R. MacGillivray, A. V. Tivanski, *J. Mater. Chem. A* **2022**; b) C. Karunatilaka, D.-K. Bučar, L. R. Ditzler, T. Friščić, D. C. Swenson, L. R. MacGillivray, A. V. Tivanski, *Angew. Chem., Int. Ed.* **2011**, *50*, 8642-8646; c) K. M. Hutchins, T. P. Rupasinghe, S. M. Oburn, K. K. Ray, A. V. Tivanski, L. R. MacGillivray, *CrystEngComm* **2019**, *21*, 2049-2052; d) T. I. Lansakara, F. Tong, C. J. Bardeen, A. V. Tivanski, *Nano Lett.* **2020**, *20*, 6744-6749.

[5] G. M. Sheldrick, *Acta Crystallogr., Sect. A: Found. Crystallogr.* **2015**, *71*, 3-8.

[6] G. M. Sheldrick, *Acta Crystallogr., Sect. C: Struct. Chem.* **2015**, *71*, 3-8.

[7] O. V. Dolomanov, L. J. Bourhis, R. J. Gildea, J. A. Howard, H. Puschmann, *J. Appl. Crystallogr.* **2009**, *42*, 339-341.
